# The practice of active patient involvement in rare disease research using ICT: experiences and lessons from the RUDY JAPAN project

**DOI:** 10.1186/s40900-021-00253-6

**Published:** 2021-02-01

**Authors:** Nao Hamakawa, Atsushi Kogetsu, Moeko Isono, Chisato Yamasaki, Shirou Manabe, Toshihiro Takeda, Kazumasa Iwamoto, Tomoya Kubota, Joe Barrett, Nathanael Gray, Alison Turner, Harriet Teare, Yukie Imamura, Beverley Anne Yamamoto, Jane Kaye, Michihiro Hide, Masanori P. Takahashi, Yasushi Matsumura, Muhammad Kassim Javaid, Kazuto Kato

**Affiliations:** 1 Department of Biomedical Ethics and Public Policy, Graduate School of Medicine, Osaka University, Suita, Osaka, Japan; 2 Department of Medical Informatics, Graduate School of Medicine, Osaka University, Suita, Osaka, Japan; 3 Department of Dermatology, Graduate School of Biomedical and Health Sciences, Hiroshima University, Hiroshima, Japan; 4 Department of Functional Diagnostic Science, Graduate School of Medicine, Osaka University, Suita, Osaka, Japan; 5 Nuffield Department of Orthopaedics, Rheumatology and Musculoskeletal Sciences, University of Oxford, Oxford, UK; 6 HeLEX Centre, University of Oxford, Oxford, UK; 7 HAEJ, Non-profit Patient Organization for Hereditary Angioedema in Japan, Kakogawa, Hyogo Japan; 8 HAEi, Non-profit International Patient Organization for Hereditary Angioedema registered in the US, Fairfax City, Virginia USA; 9 Graduate School of Human Sciences, Osaka University, Suita, Osaka, Japan

**Keywords:** Rare diseases, Patient involvement, Patient-centered research, Patient reported outcome measures, Information technology, Information and communication technology, Dynamic consent

## Abstract

**Background:**

The role of patients in medical research is changing, as more emphasis is being placed on patient involvement, and patient reported outcomes are increasingly contributing to clinical decision-making. Information and communication technology provides new opportunities for patients to actively become involved in research. These trends are particularly noticeable in Europe and the US, but less obvious in Japan. The aim of this study was to investigate the practice of active involvement of patients in medical research in Japan by utilizing a digital platform, and to analyze the outcomes to clarify what specific approaches could be put into practice.

**Methods:**

We developed the RUDY JAPAN system, an ongoing rare disease medical research platform, in collaboration with the Rare and Undiagnosed Diseases Study (RUDY) project in the UK. After 2 years of preparation, RUDY JAPAN was launched in December 2017. Skeletal muscle channelopathies were initially selected as target diseases, and hereditary angioedema was subsequently added. Several approaches for active patient involvement were designed through patient-researcher collaboration, namely the Steering Committee, questionnaire development, dynamic consent, and other communication strategies. We analyzed our practices and experiences focusing on how each approach affected and contributed to the research project.

**Results:**

RUDY JAPAN has successfully involved patients in this research project in various ways. While not a part of the initial decision-making phase to launch the project, patients have increasingly been involved since then. A high level of patient involvement was achieved through the Steering Committee, a governance body that has made a major contribution to RUDY JAPAN, and the process of the questionnaire development. The creation of the Patient Network Forum, website and newsletter cultivated dialogue between patients and researchers. The registry itself allowed patient participation through data input and control of data usage through dynamic consent.

**Conclusions:**

We believe the initial outcomes demonstrate the feasibility and utility of active patient involvement in Japan. The collaboration realized through RUDY JAPAN was enabled by digital technologies. It allowed busy patients and researchers to find the space to meet and work together for the Steering Committee, questionnaire development and various communication activities. While the practice of active patient involvement in Japan is still in its early stages, this research confirms its viability if the right conditions are in place. (331 words).

## Plain English Summary

In a number of countries, including the US, the UK and even to some extent in Japan, patients are being involved in medical research in various ways. In this research project, we were specifically interested in getting patients actively involved so that they could take part in designing and running the research project. We did this by using digital technologies, which gave us new opportunities for patients and researchers to work together as a team.

We launched an online interactive rare disease research project, called RUDY (the Rare and Undiagnosed Diseases Study) JAPAN, which was based on a similar project being carried out in the UK. RUDY JAPAN allows us to collect patient reported data, as well as to work together to design and create new ways of carrying out research through the Internet. This paper describes how we adapted the systems and concepts that were originally developed in the UK to suit the Japanese context. We also report on the following activities that enabled us to actively involve patients.

- the Steering Committee, where patients and researchers meet regularly online to discuss and make decisions about the project
- the co-creation of a new questionnaire for recording symptoms and effects of treatments
- the Patient Network Forum, where patients and physicians can connect and communicate about broad topics including quality of life issues

Through this collaboration, we demonstrated that active patient involvement with medical research using a digital platform can be put into practice in Japan. We were able to try out pre-established and new ways of working with patients. This led to effective partnership building between patients and researchers. (250 words).

## Introduction

### Patient involvement – a new trend in medical research

The role of patients and the public in medical research has changed dramatically in recent years with greater involvement and this trend will increase in importance in the future [1]. Patients are experts in their own disease experience and thus have unique perceptions and perspectives to contribute to research [2]. INVOLVE, which was established in the UK to support active involvement in the National Health Service (NHS), defines public involvement in research as being carried out ‘with’ or ‘by’ members of the public rather than ‘to’, ‘about’, or ‘for’ them [3]. Patient involvement in the medical research process is expected to enable more meaningful research that is relevant to patients’ needs [4, 5]. The focus on patient involvement is particularly noticeable in the US and the UK, with dramatic increases reported since the end of the 1990s [1].

One particularly noteworthy trend can be found in the activities of rare disease patient groups. Some groups are driving research by establishing disease-specific foundations, biobanks and/or registries to support biomedical research and conducting biomedical research on the basis of their own initiative [6]. In the context of government policy, patient-centered outcomes research [7, 8] and patient focused drug development [9] are being promoted to provide better information for decision-making in health research in the US. Similarly, the need for citizens’ direct involvement in the management of the NHS has gained recognition and patient and public involvement (PPI) is being actively promoted in the UK [3, 10].

In Japan, the number of reported cases of patient groups being involved in the planning of medical research are limited [11]. Since 2018, the Japan Agency for Medical Research and Development (AMED), a major government funding body for medical research, has started promoting PPI [12]. Given these circumstances, there seems to be a growing recognition in Japan of the need for medical research in which patients are actively involved.

### The application of ICT in the medical field

Another notable trend in medical research is the widespread use of information and communication technology (ICT), such as smartphone applications, wearable devices and interactive websites. Its application in the medical field has brought various benefits. First, ICT enables patients and citizens to participate in medical research free of temporal and spatial constraints. The widespread introduction of smart devices is expected to further lower some of the physical barriers of participation in medical research [13]. ICT-enabled research will be particularly effective for rare diseases, for which data needs to be collected from a larger geographic area than with other more common diseases if a sufficient number of patients are to be recruited to a study. Second, digitization has enabled easier storage, management and transmission of data, which makes it possible to share data quickly and widely. Third, ICT has made it possible to collect medical health information, such as daily vital signs and quality of life (QOL) data. In medical research that sets out to collect routine data, such as daily symptoms and QOL, the need for frequent input is more effective using smart devices than with paper based tools [14]. Finally, the newly developed model of dynamic consent, an interactive online consent model, provides a personalized digital interface for engaging individuals about the use of their information. It allows participants to choose and alter their consent choices over time [15].

### The beginning of RUDY JAPAN

Within these contexts mentioned above, several medical studies have been launched that use ICT to facilitate the active involvement of patients [16]. The Rare and Undiagnosed Diseases Study (RUDY) is a pioneering project that was developed by a team at the University of Oxford and launched in 2014. RUDY is an online platform that collects patient reported outcome (PRO) data including QOL, pain and functional outcomes and employs dynamic consent [17]. Patients have been encouraged “to be actively involved at all stages of the project’s development” [18]. Although RUDY in the UK (RUDY UK) initially focused on patients with rare diseases of the bone, joints and blood vessels, the project subsequently received ethical approval to include all rare diseases. As of April 2020, 2708 patients have registered [19].

In contrast to the trend in the US and the UK, there have been few such projects in Japan. It was unclear what kinds of active patient involvement and patient-researcher partnerships would be possible in Japan, and more importantly, how these endeavors should be adapted to reflect differences in ethics, regulatory and cultural aspects. There was an existing collaboration in the related field of ethical, legal and social implications (ELSI) of medical research between the University of Oxford and Osaka University. Based on this collaboration, we initiated the present research project by developing a version of RUDY in Japan (RUDY JAPAN). RUDY JAPAN has two different study aspects: medical research on rare diseases, and the practice of active involvement. In this paper, we focus on the latter aspect of the RUDY JAPAN project.

### Objective

The objective of this study was to investigate the practice of active involvement of patients in medical research in Japan through the modification of a rare disease research platform that utilizes ICT. We analyzed the processes involved and experiences of patients and researchers in order to clarify what specific approaches can be effective in the Japanese context. This paper describes how concepts and systems developed in the UK were used to create RUDY JAPAN, and reflects on the activities we have practiced: the Steering Committee, questionnaire development, dynamic consent, and other communication strategies, in terms of how to actively involve patients in medical research. We conclude with a consideration of the implications of our findings for patient-centered research more generally.

## Materials and methods

### Collaboration between Japan and the UK

Collaborative research between members of the teams at the University of Oxford and Osaka University predated the RUDY JAPAN project. A three-year project started in the spring of 2014 concerning the governance of patient-driven medical research with funding for the promotion of international collaborations from Osaka University. A questionnaire survey conducted in January 2015 as a part of this project revealed that there was a clear interest in using ICT to engage in medical research among Japanese patients [20]. In February 2015, a research member from the University of Oxford visited Osaka University and introduced the RUDY project, which was in the preparatory stage in the UK. At that time, it was decided to begin the RUDY JAPAN project. After completing a license agreement, in August 2015 the English version of the RUDY software was shared with the team at Osaka University. In November 2015, Jane Kaye, Kassim Javaid and Joe Barrett visited Osaka University for a symposium organized by Osaka University. During the symposium, the plan for creating RUDY JAPAN was publicly announced for the first time.

A Japanese version of the software was fully implemented in December 2017 after 2 years of preparation. This project had been mainly funded as exploratory research by the Japanese public funding agency, Japan Society for the Promotion of Science (JSPS) since 2015. It was initially planned for RUDY UK and RUDY JAPAN to use the same software system continuously. However, it soon became obvious that translating the content of RUDY into Japanese was a rather laborious task and the team decided that it made more sense for the Osaka side to separately construct functionality.

### Overview of RUDY JAPAN

The original RUDY study ICT platform was provided by the RUDY team in the UK, translated into Japanese first by an automatic language exchange package and then adjusted manually. Careful manual adjustments were required because of the differences in the formats of basic information (such as formats of postal codes, telephone numbers, etc.) between the UK and Japan.

The difference in ethical requirements was another factor that we needed to address. RUDY UK has used an online check box system for the consent process and has not required a handwritten signature on the consent form. In contrast, for RUDY JAPAN, we have been required to obtain the participant’s handwritten signature on the consent sheet. For this reason, we had to modify the registration system.

**Fig. 1:**
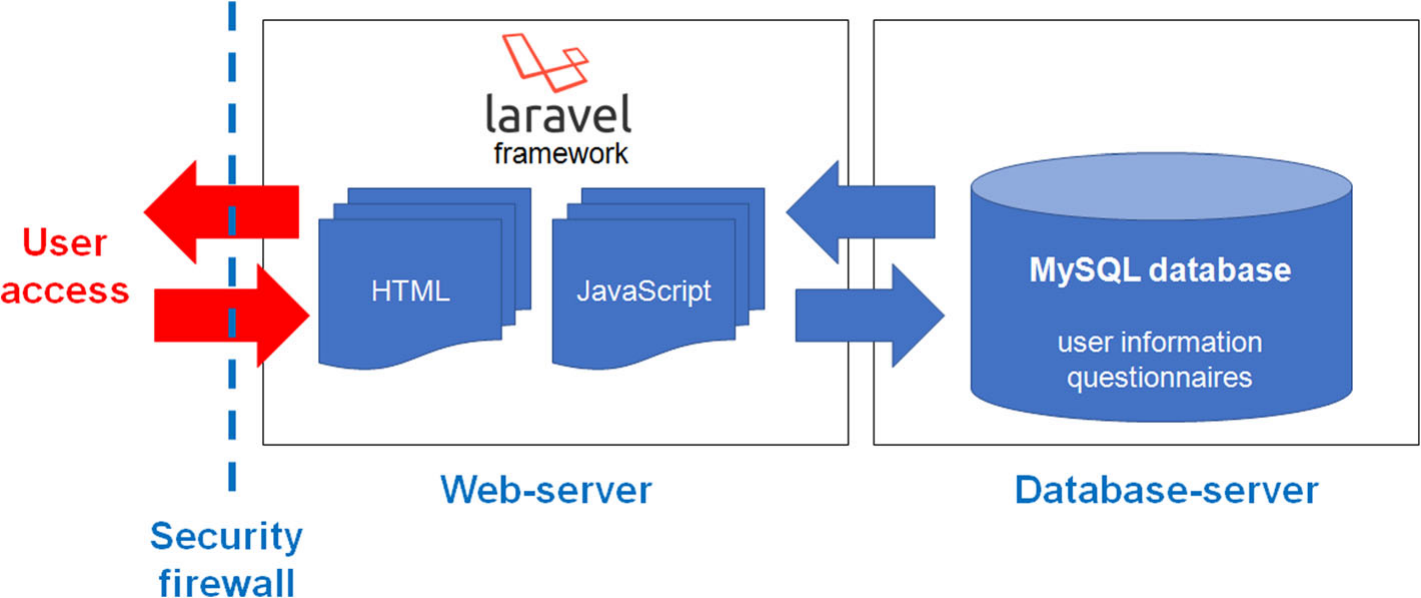
System overview of RUDY JAPAN. The figure illustrates the RUDY JAPAN system overview. The system consists of web and database servers with a high security firewall

The RUDY JAPAN system consists of two servers: the web server based on the JavaScript/PHP framework Laravel and the database server using MySQL. The servers are highly secured by Osaka University Hospital’s firewall. The system overview is shown in Fig. 1.

**Fig. 2:**
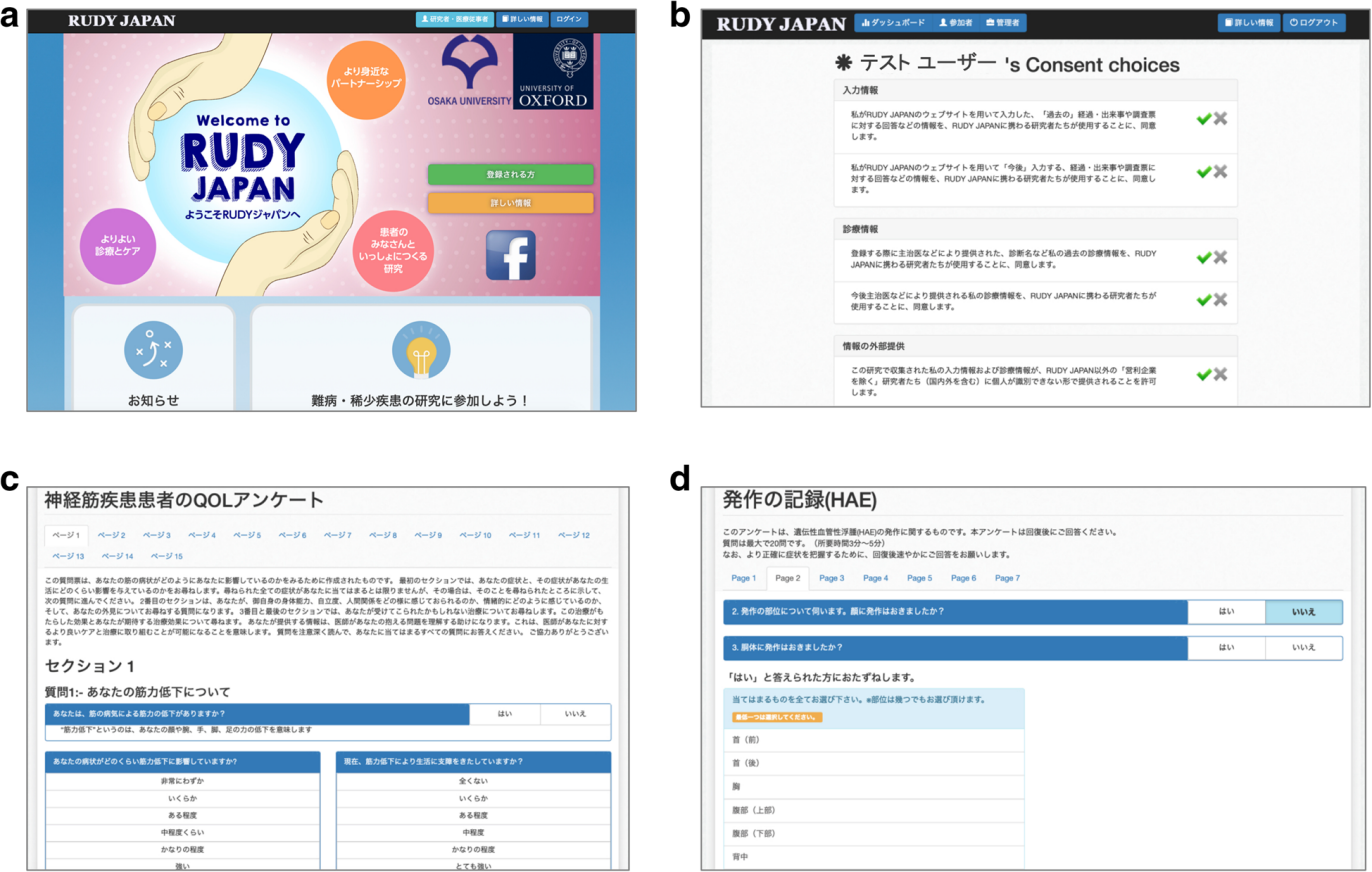
Sample views of RUDY JAPAN. The sample views of top page (**a**), the dynamic consent page (**b**) and sample questionnaire pages for the neuromuscular diseases [INQOL] (**c**) and the hereditary angioedema [HAE attack record] (**d**) are shown [21]

RUDY JAPAN web-based interfaces consist of several views: the top page including the documentation pages, the registration pages, the user information pages and the questionnaires. The questionnaires were specifically developed and/or adjusted for the RUDY JAPAN system. Sample views and the list of the questionnaires are shown in Fig. 2 and Table 1, respectively. RUDY JAPAN is freely accessible online [21]. Table 1 shows whether these questionnaires have been validated with the Japanese population or not.

**Table 1:** List of questionnaires in RUDY JAPAN

No.	Disease	Name of questionnaire	No. of questions	Type	Ref.
1	Skeletal muscle channelopathies	Severity of periodic paralysis^a^	2	Not validated	
2		Severity of myotonia	5	Not validated	[22]
3		EuroQoL (EQ5D)	6	Validated	[23, 24]
4		Epworth Sleepiness Scale (EPWORTH)	8	Validated	[25, 26]
5		Barthel index (BARTHEL)	10	Validated	[27]
6		36-Item Short Form Health Survey (SF36v2)	36	Validated	[28, 29]
7		Individualized Neuromuscular Quality of Life (INQOL)	61	Validated	[30, 31]
8	Hereditary angioedema (HAE)	Angioedema Quality of Life Questionnaire (AE-QoL)	17	Validated	[32]
9		HAE attack record	21	Newly developed	–

^a^This questionnaire was developed by a Japanese research group, Research on Rare and Intractable Neuromuscular Diseases, supported by the Ministry of Health, Labour and Welfare, Japan (https://www.nanbyou.or.jp/entry/4529)

Patients register to take part in the study via the study website, which can be accessed from PC or smart devices. We asked physicians to give patients a leaflet to introduce RUDY JAPAN. In addition, we requested that information about the study was posted on the patient group website for the target diseases and on their Facebook pages.

As of April 2020, our first cut off point for analyzing patient involvement, 58 patients had applied to register for RUDY JAPAN and 23 have completed the registration process.

### Target diseases

Target diseases of RUDY JAPAN are shown in Table 2. Skeletal muscle channelopathies were initially selected as the rare diseases that would be included in the study in December 2017. The reasons for this selection were that (1) the research collaborators, who have a background in neurology, were familiar with the relevant clinical practice in Japan and were well-positioned to cooperate with clinicians in this area and (2) there were clear benefits for both patients and researchers of these diseases to be involved in medical research with ICT because these rare diseases have very small populations and there are currently no patient groups operating in this area.

**Table 2:** Target diseases of RUDY JAPAN

**Skeletal muscle channelopathies**: two categories of rare disease	
- **periodic paralyses**: causes episodic muscle weakness [33, 34]	
- **non-dystrophic myotonias**: causes muscle stiffness (myotonia) [35].	
**Hereditary angioedema (HAE)**: a rare disease that causes unpredictable swelling attacks in various parts of the body, including the upper airway, abdomen, and outer limbs (which involves multiple disciplines of medicine) [36].	

Hereditary angioedema (HAE) was added as another target disease in October 2018. This inclusion was because of an existing connection between the patient group and the project members. The patient group was already searching for a registry platform to record data to better understand attack patterns, treatment efficacy and the disease burden on QOL. The RUDY JAPAN system, which uses ICT, was expected to enable them to collect data to capture attack patterns and impact through PRO rather than physician-reported surveys.

### Patient involvement

Several involvement approaches were designed through patient-researcher collaboration. Some were modified from the RUDY UK experience, while others were innovations of the RUDY JAPAN project.

There were two main categories of patients in this project. The first were patient partners involved in the steering of the project. The second were patient participants who registered with the RUDY JAPAN system and controlled their data use (see “dynamic consent” below).

When we started RUDY JAPAN, there were no formal guidelines about remuneration for patient involvement in medical research of this nature in Japan. Patient involvement in RUDY JAPAN has been on a purely voluntary basis with no remuneration. Given international guidelines around patient involvement, we may reconsider this as we move ahead with this project.

The main data sources for our analysis of patient involvement were minutes of the meetings (the Steering Committee, questionnaire development and various communication strategies) and the records of the system (dynamic consent). We also reflected on the diverse experiences of patients and researchers. Overall, we analyzed our practices focusing on how each approach affected and contributed to the research project. This analysis was mainly conducted by members of the Research Management Group which includes one of the patient authors (BY), a professor of sociology.

#### The steering committee

In the UK, patient input from the Patient Forum, the name given to their steering committee, has had a direct influence on decisions regarding the research, including the creation of the project name and logo and the addition of questionnaires such as sleep quality [18]. We also decided to set up a similar platform to encourage patient involvement in the form of the Steering Committee.

The patient partners of the Steering Committee were recruited through introductions by physicians or patient advocacy groups. We also recruited patients through the information website and newsletters. Potential participants were asked if they would like to contribute to the project through the Steering Committee. The value and nature of their expected contribution was explained, as well as the timing of meetings and method (every 2 months, and via an online platform).

Initially we did not recognize the need for training programs for new members of the Steering Committee, but we subsequently started to offer an orientation as well as individual support for members if and when desired.

We analyzed our experiences and comments from the patient partners focusing on how much the Steering Committee could affect the research project.

#### Questionnaire development

For skeletal muscle channelopathies, pre-existing questionnaires about QOL and symptoms of neuromuscular diseases were employed. However, no pre-existing questionnaire for HAE attacks was readily available. Although there are standard methodologies for PRO development [37], a pilot survey was necessary to grasp individual differences in symptoms and therapeutic effects that patients were aware of. Furthermore, we thought this would be a good example of collaboration between patients and researchers, which is the key concept of this project. Therefore, we developed a questionnaire in collaboration with patients to conduct an exploratory study on HAE attacks. As the target diseases are different for RUDY UK compared with RUDY JAPAN, and the UK team had not yet needed to develop a questionnaire from scratch, the RUDY JAPAN team faced a new challenge.

Development of the questionnaire was initiated following the Rare Disease Day event that was held in Kobe in February 2018, at which the RUDY JAPAN team met members of the HAE patient group.

#### Dynamic consent

RUDY JAPAN adopts dynamic consent so that participants can tailor and manage their own data sharing preferences. The contents of dynamic consent in RUDY JAPAN comprised eight items concerning data sharing preferences and six items about the way information would be communicated (Table 3). The informed consent process is currently not fully online because the first informed consent procedure is conducted via a pdf download that is completed offline then posted or scanned and submitted electronically. This reflects a key difference between the ethical requirements in the UK vs. Japan. Participants can access and change their preferences later as part of the dynamic consent approach using the secure personalized web page.

**Table 3:** Dynamic consent content

**Data use preferences**	
Primary data use	
- data that have already been submitted	
- data that will be submitted	
Clinical data (e.g., diagnosis, laboratory data)	
- data that have already been submitted	
- data that will be submitted	
Secondary data use	
- by researchers in non-profit institutions	
- by researchers in for-profit institutions	
Links to other data	
- to other familial data	
- to the data collected in other research	
**Contact from the office**	
Notification about questionnaire assignment	
Dissemination of research progression	
Re-contact for ethically approved studies	

#### Communication strategies

As many patient groups use Facebook to share their information, we created a Facebook page for RUDY JAPAN to update participants about the study and provide reminders to revisit the website.

The ‘RUDY JAPAN info’ and newsletter are two other ways we used to engage with research participants in Japan. RUDY JAPAN info provides basic information about the research, guidance on registration and updated information about activities and usage of the RUDY JAPAN website. Additionally, research progress reports and participants’ messages can be shared in a newsletter once every 6 months.

## Results

In this section, we describe the results and analyses of our practices of active patient involvement. Table 4 shows the overview of various aspects of the research process and the degree of the patient involvement based on our self-reflection. According to our assessment, patients were actively involved in many of the research processes, while in some steps, the degree of involvement was partial. In the initial phase, namely early conceptualization, system development and the first decision on the target disease (channelopathies), patients were not involved. As we moved to develop the ideas, one of the patient authors (BY) shared her experiences and expertise to enrich the concept of patient involvement.

**Table 4:** Status of patient involvement in each research process

Research process	Status of patient involvement
Conceptualization	Partially: Patients were not involved in the initial conceptualization. From 2017, one of the patient authors (BY) became more centrally involved with the project and worked to strengthen the concept.
Governance	Partially: In the initial phase, patients were not involved in the decision making. Nevertheless, we quickly established the Steering Committee and, thereafter, patients were involved in many activities including decision making and feedback.
System development	Partially: The initial software was developed in the UK and adopted to Japan by the researchers. Subsequently, the Steering Committee gave considerable feedback to improve it.
Choice of target diseases and questionnaires	Partially: Patients with channelopathies were not involved in the decisions on the target disease and questionnaires. HAE was added as a target disease based on the connection with and involvement of patients, the patient organization and researchers.
Questionnaire development	Highly: An HAE questionnaire (attack record) was developed in collaboration with patients.
Recruitment	Partially: Patient partners were involved in recruiting patient group members and suggested effective recruitment strategies.
Control of individual data use	Highly: RUDY JAPAN employs dynamic consent which allows participants to tailor and manage their own data sharing preferences.
Analysis and interpretation of the data collected through the questionnaires on the system	Not yet, but we are exploring how to conduct analysis with patients.
Communication including dissemination of the progress and results of the research	Highly: We developed a variety of communication strategies with patient partners including the Patient Network Forum, the website and newsletters.

Here, we focus on our practice that achieved a high level of active patient involvement: governance, the Steering Committee, questionnaire development, dynamic consent and other communication strategies.

### Governance

**Fig. 3:**
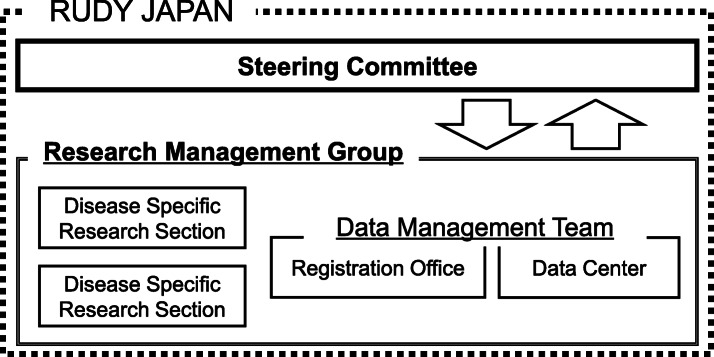
RUDY JAPAN governance structure. Patients and researchers are members of the Steering Committee. The issues or proposals that emerge from the Steering Committee are presented to the Research Management Group for consideration and implementation. Data Management is conducted by the Registration Office and the Data Center. The Disease Specific Research Sections comprise medical researchers who are responsible for each study

One initial challenge to run the RUDY JAPAN project was to establish an appropriate governance mechanism. After extensive discussions within the project team, we came up with the governance structure shown in Fig. 3. The Steering Committee provides the space for patient partners and researchers to discuss the design and management of the platform. As the project progressed, the role of the Steering Committee expanded to include making policy decisions regarding research in which patient insight would be particularly important. It is hoped that eventually the Steering Committee will make all policy decisions as the project progresses.

The Research Management Group has responsibility for day to day running of the project as well as the practical role of implementing decisions made by the Steering Committee and dealing with any technical issues. The Research Management Group comprises researchers with a variety of backgrounds: medicine, ELSI, human sciences, and informatics. The Disease Specific Research Sections, which comprise medical researchers, are in charge of direct recruitment and indirect recruitment through experts not involved in RUDY JAPAN and will be responsible for data analysis in the future. Registration of participants and allocation of questionnaires are handled by the Registration Office, which comprises researchers of ethics and governance, who contribute to the governance of RUDY JAPAN. Data management is conducted by the Data Management Team, which comprises the Registration Office and the Data Center. The Data Center comprises ICT experts. If necessary, the Research Management Group consults with researchers at the University of Oxford who developed the original RUDY UK system. This sharing of experience between the UK and Japanese teams is a unique feature of these projects and is hugely beneficial for both sides.

### The Steering Committee

The Steering Committee meetings have been held once every 2 months using a web meeting system (Zoom). In the period November 2017 to April 2020, a total of 13 meetings were held, and 11 patients and 8 researchers participated in at least 1 meeting. The Steering Committee meetings were relatively informal and allowed members to participate depending on their health condition or availability. The membership itself has increased over the current lifetime of the project. The current structure allows flexibility and long-term sustainability.

Here, we report on the results of an analysis of the Steering Committee meetings held between November 2017–February 2019. In many cases, suggestions and decisions made by patient partners in the Steering Committee have led to changes in the methods of management and platform design in RUDY JAPAN. The issues that have been discussed during the initial nine meetings are shown in Table 5. Of the 16 comments, 11 changes have already been implemented or are currently in progress. For instance, to reduce the time it takes to register, an envelope has been enclosed with a consent sheet and research participants who are in the process of completing their registration can now respond immediately by sending an email to support registration. Additionally, frequently asked questions, including research objectives and benefits, are summarized and were made accessible at any time under “RUDY JAPAN info”. Furthermore, patients expressed their desire to connect with doctors and other patients and share information related to their disease, which later led to the establishment of the “Patient Network Forum” described below.

**Table 5:** Changes in research design and management made in response to the Steering Committee

No.	Topic	Patient comments	Status A: response completed B: in progress C: under consideration D: unchangeable
1	Study benefit	The benefits of participation in research are unclear.	A
2	Study purpose	The purpose of the study is unclear.	A
3	Research progress	The progress of the research should be disseminated.	A
4	Patient needs	An opportunity to connect with other patients and doctors and better understand diseases is important for patients.	A
6	Registration process	The registration pathway is time-consuming.	C
		Return envelope for sending the consent form should be attached when participants are requested to mail a consent sheet.	A
		Explanation of the consent sheet format is unclear.	A
7	Participant reminders	The provisional registration reminder should be sent earlier.	A
		People who have not responded to the questionnaire should be reminded.	C
8	Wording difficulties	The expressions used in the questionnaires are difficult to understand.	D
9	Volume and description of questionnaires	The amount covered in questionnaires is too burdensome.	D
		The required range of responses to the question is unclear.	B
		The approximate time to complete each questionnaire should be noted.	B
10	Meeting names	The name of the meeting should be changed to suit the purpose.	A
11	Social media	Images and links should be added to the article.	A
		Information on symptoms and other studies will increase the value of the article.	C

The table shows the changes to the study management / design suggested by patients in the initial nine meetings (November 2017 – February 2019). The comments can be divided into 11 categories. Each letter indicates the comment status; A: response completed, B: in progress, C: under consideration, D: unchangeable. Of the 16 comments, 11 have already been implemented or are currently in progress

The Steering Committee discussions have increased the number of issues that we have been able to identify and address. Therefore, patient partners and researchers have also considered and decided which issues should be prioritized.

As Table 5 demonstrates, it was difficult to respond to some of the patients’ opinions. Most notably, several patients suggested that they would like to change the wording of the questionnaires. However, most of the questionnaires have been validated by other research groups; therefore, it was important that the original wording was retained. Also, the volume of questionnaires could not be reduced because of the research purpose. Nevertheless, because these issues were raised in the Steering Committee meeting, we addressed them and better explained the researcher perspective.

A final issue raised in the meetings was individual differences in patients’ motivation to participate in the research. Participation in the Steering Committee requires the commitment of time, often outside of normal office hours. Patient partners and researchers needed to allocate time for the meetings every 2 months. To maximize participation, the meetings were generally held during the evening on weekdays or weekends. This could potentially present a challenge to the sustainability of the Steering Committee's activities.

### Questionnaire development

**Fig. 4:**
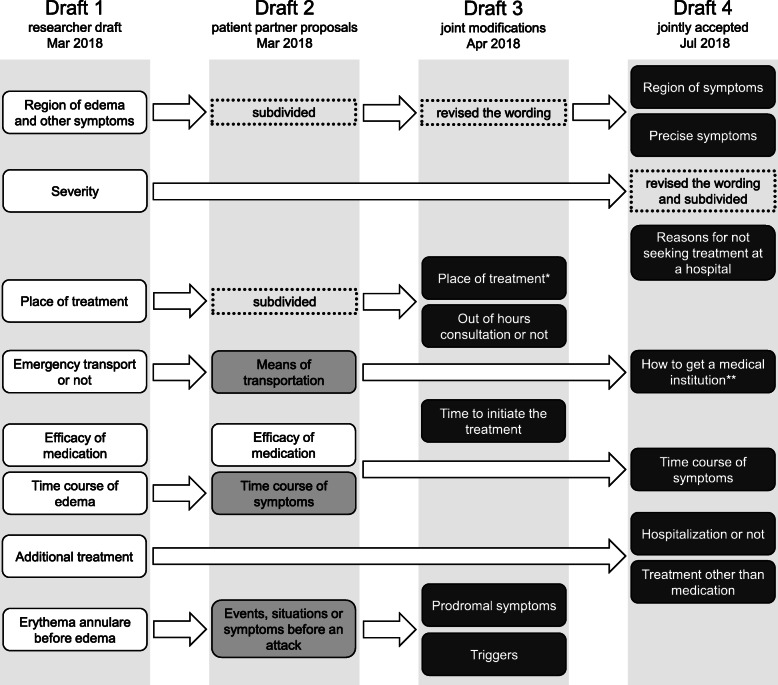
Questionnaire development process. Only the items of the questionnaire where contents were changed are shown in this figure. The first draft was made by the researchers. The second draft reflects the patient partner’s opinions on the first draft. The third draft was developed through the first online meeting, which was attended by both the patient partner and researchers. An independent review by medical researchers and subsequent online meetings resulted in the final draft. The items in the black box are the fixed questions. *This item includes medical institutions and home. **This item includes whether the patient visited a medical institution by him/herself, with someone’s help or by ambulance

The questionnaire development process is summarized in Fig. 4. First, the medical researchers prepared a draft questionnaire (Draft 1) and then revised this based on comments from one of the patient partners (Draft 2). At that time, requests were made to investigate the body region where the symptoms presented, the place of treatment and any possible triggers for the attack in more detail. In the first online meeting, the importance of the time from an attack to receiving treatment and the need to distinguish between prodromal symptoms and attack triggers were discussed (Draft 3). Following this, the questionnaire was independently reviewed by medical researchers who are not HAE specialists, who provided advice on including “N/A” as an option and on improving potentially conflicting questions, such as “time to onset of symptomatic improvement after treatment initiation” and “degree of improvement an hour after treatment initiation.” Five subsequent online meetings and email discussions followed, which included discussions about how to organize questions, where attacks occurred, how patients travel to a medical institution for treatment and the course of symptom development over time. Questions were developed not only by refining the structure of the proposed questions, but also by adding new questions that had not previously been considered. For example, a question asking why a patient did not visit a hospital for treatment during an attack had not initially been proposed by either patients or researchers, but was jointly developed through a discussion between the two groups. This question was initially proposed after a patient partner suggested that seeking or not seeking treatment during an attack is not necessarily associated with severity of symptoms (Draft 4).

During the questionnaire development, it took time to build a consensus on some of the questions because of difficulties in understanding different stakeholder perspectives. Especially for patients without academic training, the rigorous discussion about scientific validity of the questionnaire appeared demanding on occasions. Therefore, it took some time and patience to both reflect patient perspectives and ensure scientific validity.

### Preliminary dynamic consent results

The registration office contacts participants based on their preferences. Regarding preferences for being notified about the study, 12 (71%) of 17 who completed the registration chose email, whilst 5 (29%) chose a letter. Concerning the method of notification, 13 (77%) participants wanted to be notified to complete a new questionnaire by email and 4 (23%) wanted to be notified by letter. No participants selected contact by telephone. To date, none of the participants have changed their consent settings including data usage.

The RUDY JAPAN team adapted the consent form used by RUDY UK to suit the study needs. The consent process for RUDY UK includes consent decisions relating to the provision of biospecimens for storage and future research. However, there is no intention to collect biospecimens in Japan at this time. Therefore, these sections of the consent form were removed for RUDY JAPAN. Moreover, during the translation process, expressions that did not adequately reflect the situation in Japan or that might not make sense to Japanese patients were changed and consent items regarding data sharing and links to other studies were modified.

### Various communication strategies

Table 6 shows the practical methods for communicating with participants over time, in addition to the Steering Committee and questionnaire development.

**Table 6:** Various communication strategies

	Dates	Main purposes
RUDY JAPAN Facebook (public) [38]	Since Dec. 2017	To disseminate information daily and externally. Post images and videos, share links.
RUDY JAPAN info (public) [39]	Since Dec. 2017	To provide research information and update activities mainly for prospective and current participants, in addition to guide how to register and use RUDY JAPAN.
Newsletter (public)	Oct. 2018, Mar. 2019	To periodically report progress in the development of the platform, as well as research results and messages from researchers and patients to participants.
Patient Network Forum (semi-private)	Jan. 2019 Oct. 2019	To facilitate communication and networking among patients and between patients and doctors. The main topics include daily lives of patients and questions about clinical care.

RUDY UK demonstrated significant benefits from using social media to increase the study’s external profile and improve recruitment. Similarly, our Facebook page allowed RUDY JAPAN to post images and share links, and helped to engage with participants as well as public in the work we are doing.

The RUDY JAPAN info (the information website) and newsletter are the examples where we have improved on the RUDY UK model. We have built on the design of the website over time, based on the discussion with patients in the Steering Committee. Another notable finding is that the some of the patient participants responded to the newsletter by emailing their opinions to help improve the project.

The idea of the Patient Network Forum was emerged from the discussion with patient partners in the Steering Committee. There was a need of patients to connect with other patients and physicians. Unlike the Steering Committee which focuses on decision-making for the research project, the Patient Network Forum aimed to facilitate networking and communication about broad topics including patients’ daily lives. The Patient Network Forum used an online meeting system (Zoom). Patients were invited to join this Forum through several avenues, namely social media, emails to patients who had already registered with RUDY JAPAN, and introductions by existing patient members of the Steering Committee. Patients and researchers worked together to organize the forum. On the first forum, five patients and four researchers, including two disease specialists, discussed different aspects of the disease and shared patient experiences. The follow-up questionnaire contained positive comments from all patients, such as “I am glad that I had the opportunity to share stories about various health conditions.”

## Discussion

In this paper, we have detailed our experiences implementing RUDY JAPAN as an example of patient-centered medical research using ICT. Patient involvement has yet to be practiced widely in medical research in Japan, despite various studies calling for active involvement of patients [40, 41]. While recognizing varying levels of involvement, here, we define active involvement as patients playing a key role in research, which is largely consistent with the definition by INVOLVE. To the best of our knowledge, this paper is the first report on this practice in Japan. Although the RUDY JAPAN project has just completed the first stage of establishing patient-researcher partnerships and recruitment of patients is still ongoing, we believe that our experiences and lessons from the collaboration can contribute to the emerging field of patient involvement in medical research.

### Lessons from collaboration between the UK and Japan

RUDY JAPAN demonstrates that medical research that promotes active patient involvement is viable in Japan. Most importantly, RUDY JAPAN, like RUDY UK, successfully implements patient involvement strategies that have been shown to work in other cultural settings thus confirming their veracity and applicability. Patients who participated in our project showed strong enthusiasm towards mutual collaboration with researchers.

As expected, ICT proved to be a powerful tool throughout the project. The Steering Committee meetings in Japan created a virtual space for patients to influence the design and direction of research decisions, just as the Patient Forum does for RUDY UK [18]. The ease of participation gave patients the confidence that they could regularly join meetings and contribute. In addition, we believe that the virtual meeting space reduced possible barriers that could have been constructed between patients and researchers had we met in a physical space, such as the university.

Another finding was that practical modification of RUDY system was necessary to suit the new location with its different healthcare system. As mentioned in the Materials and Methods section, during the implementation process, it was not possible to import the system wholesale from the UK to Japan. Thus, modification was required in a variety of details of software and registration procedures, to cope with the differences in healthcare structures and ethical requirements. Reflecting the connections and expertise of the researchers on the Japan side, we targeted different diseases from the UK, which again required creative adaptation.

Through the RUDY collaboration, project members learned valuable lessons for international research projects between countries with different healthcare landscapes, namely sharing concepts and overcoming practical challenges.

### Approaches to patient involvement in RUDY JAPAN

The following research activities represent varying levels of active patient involvement: the Steering Committee, questionnaire development and communication strategies. First, we describe the activities and then, we discuss the degree to which each enabled active involvement. Finally, we consider some of the possible negative aspects that this way of working presents to stakeholders.

First, the Steering Committee was essential for ensuring that both patients’ and researchers’ perspectives were being reflected in the research process. Virtual meeting tools enabled patient partners to take part in these meetings without requiring onerous time commitments or travel requirements. By holding the meetings every 2 months, discussions continued effectively to allow decisions to be fed directly into project development. Table 5 summarizes our analysis of the outcomes of patient involvement through the Steering Committee. As can be seen, the Steering Committee represents high level involvement of patients as their contribution has directly contributed to priority setting and project methodology.

Second, development of a questionnaire for HAE attack recording was a new attempt to collaborate with patients from an early stage in the research cycle. The researchers’ professional knowledge and experience, combined with the patients’ expert knowledge and experience (impact on daily life and behavior) are considered to be complementary elements [42]. We demonstrated that questionnaire development is another way to create new research frameworks that could not have been achieved by patients nor researchers working alone. We found that both patients and researchers experienced mutual learning through the interactions for questionnaire development.

Third, we clarified the importance of a variety of communication strategies. We conclude that the practices described in the results section helped patients’ understanding of the study and reduced barriers to involvement. It is notable that the Patient Network Forum cultivated dialogue between patients and physicians, which helped researchers to understand the needs and challenges of patients in their daily lives. These multiple communication channels helped to increase interactions between researchers and patients, including those who were not members of the Steering Committee. While the influence of the Patient Network Forum was indirect to the steering of the project, it had a direct impact on the reach of the project, and depth of interaction between patient participants. This novel rare disease patient network, in the context of Japan, was enabled by the RUDY ICT platform. We also plan to utilize these variety of methods to disseminate the results of the questionnaire data analyses that will be forthcoming.

Finally, while we achieved varying levels of active involvement, this is not without recognizing new challenges in this way of doing research. The process required time and effort for both patients and researchers. We had to take special care in the organization of meetings, paying attention to the length and facilitation methods, as well as taking into account the possibility that discussions with researchers may be burdensome for those patients not used to academic exchange. Nevertheless, we believe the benefits of this way of doing research outweigh the challenges.

### Implications for the success of patient-centered research

Through our work, we can discuss implications that are essential for the success of patient-centered research, which include partnership and actual benefits for patients. Interestingly, we found these concepts are shared by both RUDY JAPAN and RUDY UK.

#### Patient-researcher partnership

Partnership is essential for patient-researcher collaboration. It has been gradually generated by building trust using various forms of communication and collaboration, thereby demonstrating the trustworthiness of the research endeavor. Online communication has been proved to effectively facilitate and foster partnerships by making it easier and communicate frequently.

Throughout our practice, we found that there were differences in perspectives between patients and researchers. Both groups have learned mutually through the interactions involved in such activities as discussions that took place in the Steering Committee and the development of the questionnaire. Researchers were able to experience first-hand how patients think about research and what is important when articulating ideas about aims and benefits to the patient community. Patients were able to gain perspectives on how researchers think about their research and frame ideas. They were able to better appreciate the issues that have to be considered, such as validity, ethics and regulatory practices. Through discussions and the practice of working together new understandings and perspectives emerged that, we believe, can produce more relevant research and research outcomes.

Facilitation was a key to creating an environment in which both patients and researchers can have constructive discussions towards the overall project aims. In the future, it would be necessary to consider the background and circumstances of each partnership and find the most appropriate methods to support involvement for each individual. It will be also important to ensure that all patients including those who do not want to be extensively involved in research design, can participate in the project according to their interest and capacity.

#### Considering actual benefits for patients

During the study, project members (both RUDY JAPAN and RUDY UK) worked together to clarify the actual benefits for participants. Patients who participated in the Steering Committee also emphasized the importance of making it easier for patients to understand the benefits of participating in the research. The discussion between patients and researchers allowed the project team to recognize the diverse range of benefits, including the following: 1) Fundamental benefits: benefits as research outcomes that potentially provide better treatments or healthcare for patients, 2) Tangible benefits: benefits that meet and fulfill individual patients’ immediate needs, such as self-tracking symptoms, medication records, and patient gathering events. We can also speculate that intangible benefits accumulate. These could include self-efficacy, that patients can believe in their expertise to influence research activities, and growing familiarity with research and the research process, that allows patients to feel greater confidence to offer opinions and frame ideas.

It is crucial to continue discussions with patients on the tangible and intangible benefits that are needed or possible and how they can be realized. One key area is to invite patients who have participated at some level back to describe the types of information collected and have them discuss and decide on the priority list of analyses.

## Limitations and future challenges

While RUDY JAPAN has only been working with patients from two disease groups, we are currently expanding the disease areas and will continue to do so into the future.

Given that only a small number of patients were involved in the Steering Committee and questionnaire development, there may be a limitation in the diversity of patients at the high end of active involvement. As we have not collected data that allows us to assess socio-economic background of participants, it is hard to be definitive on this question. Clearly this is something we need to address in the future.

We are also aware of the issues related to the digital divide. We have already faced the challenge that some of the prospective participants have had difficulty to complete the registration because of their unfamiliarity with ICT. We have used other communication tools, including telephone and letters, to overcome such a challenge.

## Conclusions

This paper reported on a project that is investigating the practice of patient involvement in medical research in the Japanese context. Our approaches of active patient involvement were successfully put into practice. It was found that a high level patient involvement was achieved through the Steering Committee and questionnaire development. Various communication strategies cultivated dialogue between patients and researchers. While we cannot generalize at this stage, we believe that our experiences and lessons can provide insights for future practice.

## Data Availability

The datasets used and/or analysed during the current study are available from the corresponding author on reasonable request.

## Abbreviations

NHS: National Health Service
PPI: Patient and public involvement
AMED: The Japan Agency for Medical Research and Development
ICT: Information and communication technology
QOL: Quality of life
RUDY: Rare and undiagnosed diseases study
PRO: Patient reported outcome
ELSI: Ethical, legal and social implications
HAE: Hereditary angioedema
